# Massive acetaminophen overdose with metabolic acidosis refractory to *N*-acetylcysteine, fomepizole, and renal replacement therapy

**DOI:** 10.1016/j.toxrep.2021.03.031

**Published:** 2021-04-06

**Authors:** Sean Cuninghame, Khaled Lotfy, Paul Cameron

**Affiliations:** a Department of Medicine, Western University, London, ON, Canada; b Division of Nephrology, Department of Medicine, Western University, London, ON, Canada; c Division of Critical Care, Department of Medicine, Western University, London, ON, Canada

**Keywords:** Overdose, Acetaminophen, APAP, Renal replacement therapy, Intermittent hemodialysis, Metabolic acidosis

## Abstract

Massive Acetaminophen (*N*-acetyl-p-aminophenol; APAP) overdose is a common presentation to emergency departments around the world. While *N*-acetylcysteine (NAC) remains the cornerstone of treatment for APAP overdose, extracorporeal treatment, in the form of renal replacement therapy with intermittent hemodialysis (IHD) or continuous renal replacement therapy (CRRT) may provide benefit in cases associated with altered mental status and metabolic acidosis. One treatment with IHD is typically sufficient for resolution of acidosis and global improvement clinically. We describe a case of massive APAP overdose presenting with altered mental status and lactic acidosis, refractory to multiple treatments of IHD as well as CRRT and high-dose NAC along with fomepizole. Despite these interventions, fulminant liver failure progressed with cerebral edema, coagulopathy and death. This is the first description of a fatal acetaminophen ingestion refractory to both IHD and prolonged CRRT. This case highlights the need for further investigation in the management of massive APAP overdose, including optimal method and timing of renal replacement therapy.

## Highlights

• Acetaminophen toxicity can be associated with a metabolic acidosis and treated with Renal Replacement Therapy.
• Metabolic acidosis refractory to renal replacement therapy likely leads to worse outcomes.
• Cystatin C should be used as a marker of renal function in acetaminophen toxicity.

## 1 Introduction

APAP (*N*-acetyl-p-aminophenol; APAP) is the most widely used analgesic in the world and this widespread availability has rendered it a common agent in intentional overdose [1]. The mainstay of management of APAP toxicity is *N*-acetylcysteine (NAC), which replenishes hepatic glutathione stores, allowing for conversion of *N*-acetyl-p-benzoquinone imine (NAPQI; the toxic metabolite of APAP) to non-toxic metabolites. While numerous experimental. treatments are still being investigated to improve outcomes in APAP overdose [2], NAC treatment alone is often sufficient to prevent the development of severe toxicity. However, in cases of massive APAP ingestions (i.e., above 0.5 g/kg), early glutathione depletion leads to an overabundance of NAPQI, which may not be sufficiently converted to non-toxic metabolites with NAC alone. In these cases, early lactic acidosis (i.e., within hours to days) may result from depletion of liver glutathione stores leading to 5-oxoprolinemia, NAPQI-induced mitochondrial dysfunction, and APAP binding to mitochondrial enzymes [3,4]. In contrast, the development of late (i.e., several days after ingestion) acidosis is hypothesized to be related to reduced hepatic lactate clearance [5]. NAPQI production is also hypothesized to be the culprit of acute kidney injury (AKI) leading to renal tubular damage in many cases of APAP toxicity [6]. With early lactic acidosis, extracorporeal treatments (ECTR) such as hemodialysis may be beneficial due to the dialyzable nature of APAP. In 2014, the EXtracorpeal TReatments In Poisoning (EXTRIP) working group published guidelines on the use of extracorporeal treatments in APAP toxicity [7]. As part of their recommendations, ECTR has been recommended alongside NAC if the patient has altered mental status, metabolic acidosis, an elevated lactate, along with an APAP level greater than 5960 μM.

We describe a case of a young woman with massive APAP ingestion, early lactic acidosis and fulminant hepatic failure refractory to repeated treatment with multiple modalities of renal replacement including prolonged high-dose IHD followed by Continuous Venovenous Hemodiafiltration (CVVHDF). To the best of our knowledge, this is the first case of APAP toxicity and early lactic acidosis where repeated extracorporeal treatments were unsuccessful in elimination of the offending parent compound and prompt resolution of acidosis.

## 2 The Case

A 24-year-old woman was brought to a community hospital with decreased level of consciousness. Medical history was significant for endometriosis, chronic pelvic pain, depression with previous suicide attempts, and generalized anxiety disorder. Collateral history estimated an ingestion of 50 g of APAP, 25.5 g of gabapentin, and 60 mg of morphine sixteen hours before presenting. Initial vital signs were normal despite her reduced level of consciousness. Admission investigations were remarkable for: APAP level of 3661 μM, lactate of 3.8 mM, pH of 7.22 and bicarbonate of 9 mM. Creatinine was 56 μM. Alanine aminotransferase (ALT) and alkaline phosphatase (ALP) were 113 U/L and 64 U/L, respectively, with a total bilirubin of 10 μM and International Normalized Ratio (INR) of 1.6. NAC was initiated, with a bolus of 60 mg/kg over one hour followed by an infusion of 12 mg/kg/hr thereafter in addition to 600 mg IV fomepizole. Peak APAP level, two hours after presenting, was 3983 μM. Subsequently, the patient was intubated for airway protection and transferred to our tertiary Medical-Surgical Intensive Care Unit (ICU) for consideration of liver transplantation.

The patient arrived to our ICU thirty-two hours after ingestion. She was afebrile with a blood pressure of 110/60 mm Hg without inotropic support, a heart rate of 92 beats per minute (bpm), and oxygen saturation 94 % on pressure support ventilation with PEEP 5 cm H_2_O and pressure support of 8 cm H_2_O. Without sedation, she had spontaneous eye opening and followed commands. Pupils were equal and reactive. Cardiovascular and respiratory physical examinations were unremarkable. Laboratory data showed APAP level of 2931 μM, and lactate of 4.5 mM. Arterial blood gas (ABG) analysis revealed pH 7.24, PaCO2 of 29 mm Hg, and PaO2 of 405 mm Hg. Electrolytes were within normal limits. Creatinine was unmeasurable due to presence of NAC. ALT rose to 206 U/L with ALP of 46 U/L. INR was 3.9, with total bilirubin that rose to 19.0 μM.

**Fig. 1:**
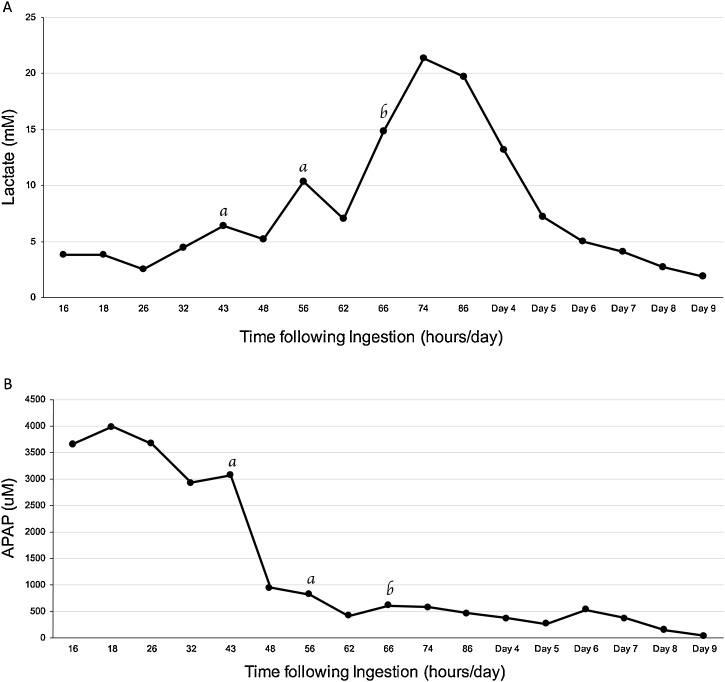
Effect of Renal Replacement Therapy on (A) Lactate and (B) APAP levels. The start of IHD (a) and CRRT (b) are indicated.

NAC and fomepizole were continued at a rate of 12 mg/kg/hr and 600 mg IV every six hours, respectively. Laboratory investigations four hours later showed worsening acidosis despite bicarbonate infusion. Therefore, IHD was initiated fifty-eight hours after ingestion. The following prescription was used: Fx1000 filter, a target blood flow (Qb) rate of 400 mL/min, with a dialysate flow of 500 mL/min and a bath of 140 mM of Sodium, Potassium 4.0 mM, Calcium 1.25 mM, Bicarbonate 40 mM. Her APAP level prior to dialysis was 3071 μM with lactate level of 6.8 mM. She received one four-hour run of IHD with a volume of blood processed (VBP) of 100.2 L, followed by a second six-hour run of IHD with a VBP of 144.4 L within a twenty-four-hour period. Despite these two runs of IHD, the lactate level rose to 14.8 mM while her APAP level remained elevated at 437 μM (Fig. 1). At this point, she was switched to CRRT due to concerns regarding the development of cerebral edema with IHD. Therefore, CVVHDF was started with a fluid removal target of 100 mL/hr, blood flow rate of 250 mL/min, a bath of sodium at 140 mM, potassium 4 mM, Calcium 1.25 mM and Bicarbonate 40 mM.

Despite ongoing CVVHDF, her APAP levels increased again to 614 μM after twelve hours of CVVHDF. Transaminases exceeded 8000 U/L, twenty-two hours after initiating CVVHDF. After forty-eight hours of CVVHDF, lactate decreased, however APAP levels were refractory, remaining elevated at 465 μM. Whole bowel irrigation and colonic decompression were attempted but unsuccessful secondary to ileus development. Not until post-ingestion day eight, and after seven days of CRRT did the APAP level become undetectable. Throughout this time, the patient developed fulminant liver failure, with coagulopathy, hypoglycemia and encephalopathy.

Computerized Tomography (CT) imaging of the head revealed widespread cerebral edema and petechial hemorrhage on post-ingestion day five. Measures to reduce cerebral edema including hypertonic saline and hypertonic dialysate targeting serum sodium of 150−155 mM were employed. Magnetic Resonance Imaging (MRI) of the brain post-ingestion day twelve showed diffuse axonal injury. Life-sustaining measures were withdrawn day sixteen after admission following discussions with family on the basis of her poor neurologic prognosis.

## 3 Discussion

To our knowledge, this is the first case reported of a fatal, massive ingestion of APAP refractory to repeated treatments utilizing multiple modalities of renal replacement therapy in the context of NAC and fomepizole co-administration. Massive APAP overdose can present with altered level of consciousness and early metabolic acidosis due to mitochondrial toxicity from NAPQI production [3]. Renal replacement therapy may be indicated in such cases to efficiently correct acidosis and reduce serum APAP levels [7]. However, NAC administration on its own is often sufficient to avoid renal replacement, as it also prevents lactate accumulation and protects against hepatotoxicity thereby supporting hepatic lactate clearance [8]. In most cases of early lactic acidosis in APAP overdose, the lactatemia persists for less than 48 h, even when renal replacement therapy is not used to mitigate the patient’s acidosis [9]. When renal replacement therapy is employed, however, APAP clearance is more efficient with a rapid improvement of the associated acidosis, typically within hours of first IHD treatment in most reports [3,4,7,10,11]. In these cases, additional runs of IHD are not necessary in order to improve the patient’s lactate, and IHD use coincides with clinical improvement as well. While our patient did not have a detectable APAP above 5960 μM as suggested by the EXTRIP guidelines, given her altered mental status and lactic acidosis, consideration of extracorporeal management was provided. In our case, renal replacement therapy was initiated with two treatments of IHD over ten hours, and thereafter switched to CCVHDF. Despite this, her lactate was not successfully cleared until after more than five days of CVVHDF (Fig. 1A), and the patient’s APAP levels remained detectable for over a week of renal replacement therapy, at times increasing from previous measurements (Fig. 1B). Despite two runs of IHD, our patient’s early lactic acidosis continued to worsen.

As with any antidote, the efficacy of NAC on preventing hepatotoxicity and its sequelae are dependent on both the timing from toxic ingestion and the dose of antidote. It is important to note that in this case, the patient presented sixteen hours after her ingestion. Ideally, NAC should be initiated as soon as possible following ingestion, as delayed NAC administration is associated with worse outcomes [12]. The delay in receiving NAC may have contributed to the poor outcome, although delayed NAC (i.e., more than 10 h from ingestion) still has mortality benefit [13] and conversely, hepatotoxicity may still develop despite early NAC initiation [14]. Furthermore, given that NAC is dialyzable, an increased dose is recommended for patients who receive IHD, but not during CRRT [7,15,16]. However, to our knowledge there is no clear consensus regarding the mechanics of NAC dosing during renal replacement. A recent case report described more than tripling a NAC doses (up to 18 mg/kg/hr while off dialysis; 36 mg/kg/hr while on dialysis) in a patient who survived a massive APAP overdose [4]. Further studies on NAC dosing and its benefit in massive APAP overdose where patients receive renal replacement therapy are needed.

Lastly, AKI is commonly associated with APAP overdose, the severity of which correlates with mortality risk [6]. Therefore, accurate measurement of renal function is essential to management. Evidence is conflicting regarding potential interference of NAC on creatinine and cystatin C assays [17,4]. In our case, creatinine was not measurable using the assay in our laboratory which uses the CREatinine Plus version 2 (CREP2) Cobas® enzymatic assay system by Roche®. The product insert describes that *N*-Acetylcysteine above serum concentrations of 333 mg/L can interfere with the assay. In addition, NAPQI itself may also interfere with enzymatic measurement of creatinine using this enzymatic method. While some cases have highlighted NAC and/or NAPQI and APAP interference with serum creatinine, this is the first case report to suggest cystatin C as the preferred method of renal function assessment APAP overdose. Our laboratory uses the Cystatin C Cobas® enzymatic assay by Roche® as well, which does not demonstrate interference with NAC and/or NAPQI. In our case, measurement of serum cystatin C was detectable despite use of NAC. As cystatin C is a reliable biomarker of AKI [18], we suggest the use of cystatin C as an alternative for monitoring renal function in patients being managed for massive acetaminophen overdose. Future studies should help validate the reliability of cystatin C as an alternative measurement of renal function specifically in APAP overdose both with and without NAC use.

## 4 Conclusion

APAP overdose is a common presentation to emergency departments around the world. NAC remains the cornerstone of management and most patients recover with NAC alone. However, in cases of massive APAP overdose, renal replacement therapy may be indicated in cases of acidosis and altered mental status. Our patient presented with early metabolic acidosis and an altered mental status that required airway protection. Her acidosis and APAP levels did not resolve despite multiple runs of intermittent hemodialysis within seventy-two hours of ingestion and several hours of CRRT thereafter alongside a continuous high-dose NAC infusion. She would eventually go on to develop fulminant liver failure with associated cerebral edema. This case prompts further investigation into the appropriate use of renal replacement therapy in cases of severe APAP overdose.

## Funding

This research did not receive any specific grant from funding agencies in the public, commercial, or not-for-profit sectors.

## Conflict of interest

The authors declare no conflict of interest.

## References

[1] Prescott K., Stratton R., Freyer A., Hall I., Le Jeune I. (2009). Detailed analyses of self‐poisoning episodes presenting to a large regional teaching hospital in the UK. Br. J. Clin. Pharmacol..

[2] Hussain S., Ashafaq M., Alsharani S., Siddiqui R., Ahmed R.A., Khuwaja G., Islam F. (2020). Cinnamon oil against acetaminophen-induced acute liver toxicity by attenuating inflammation, oxidative stress and apoptosis. Toxicol. Rep..

[3] Ghannoum M., Kazim S., Grunbaum A.M., Villeneuve E., Gosselin S. (2016). Massive acetaminophen overdose: effect of hemodialysis on acetaminophen and acetylcysteine kinetics. Clin. Toxicol..

[4] Murphy Daniel (2020). A quarter pound of acetaminophen with propylene glycol on the side: a case report. Clin. Nephrol. Case Stud..

[5] Wiegand Timothy J., Margaretten Mary, Olson Kent R. (2010). Massive acetaminophen ingestion with early metabolic acidosis and coma: treatment with IV NAC and continuous venovenous hemodiafiltration. Clin. Toxicol..

[6] Stollings J.L., Wheeler A.P., Rice T.W. (2016). Incidence and characterization of acute kidney injury after acetaminophen overdose. J. Crit. Care.

[7] Gosselin S., Juurlink D.N., Kielstein J.T., Ghannoum M., Lavergne V., Nolin T.D., Hoffman R.S., Extrip Workgroup (2014). Extracorporeal treatment for acetaminophen poisoning: recommendations from the EXTRIP workgroup. Clin. Toxicol..

[8] Eikemans B.J.W., Mauritz R., van Westerloo D.J. (2016). Early lactic acidosis after acetaminophen overdose. Netherlands J. Crit. Care.

[9] Zein J.G., Wallace D.J., Kinasewitz G., Toubia N., Kakoulas C. (2010). Early anion gap metabolic acidosis in acetaminophen overdose. Am. J. Emerg. Med..

[10] Woolum J.A., Hays W.B., Patel K.H. (2020). Use of fomepizole, n-acetylcysteine, and hemodialysis for massive acetaminophen overdose. Am. J. Emerg. Med..

[11] Kiernan E.A., Fritzges J.A., Henry K.A., Katz K.D. (2019). A case report of massive acetaminophen poisoning treated with a novel “triple therapy”: N-acetylcysteine, 4-methylpyrazole, and hemodialysis. Case Rep. Emerg. Med..

[12] Rumack B.H., Bateman D.N. (2012). Acetaminophen and acetylcysteine dose and duration: past, present and future. Clin. Toxicol..

[13] Klein-Schwartz W., Doyon S. (2011). Intravenous acetylcysteine for the treatment of acetaminophen overdose. Expert Opin. Pharmacother..

[14] Doyon S., Klein‐Schwartz W. (2009). Hepatotoxicity despite early administration of intravenous N‐acetylcysteine for acute acetaminophen overdose. Acad. Emerg. Med..

[15] Sivilotti M.L.A., Juurlink D.N., Garland J.S., Lenga I., Poley R., Hanly L.N., Thompson M. (2013). Antidote removal during haemodialysis for massive acetaminophen overdose. Clin. Toxicol..

[16] Hernandez S.H., Howland M., Schiano T.D., Hoffman R.S. (2015). The pharmacokinetics and extracorporeal removal of N-acetylcysteine during renal replacement therapies. Clin. Toxicol..

[17] Rehman T., Fought J., Solomon R. (2008). N-acetylcysteine effect on serum creatinine and cystatin C levels in CKD patients. Clin. J. Am. Soc. Nephrol..

[18] Coca S.G., Yalavarthy R., Concato J., Parikh C.R. (2008). Biomarkers for the diagnosis and risk stratification of acute kidney injury: a systematic review. Kidney Int..

